# Spread of porcine circovirus associated disease (PCVAD) in Ontario (Canada) swine herds: Part I. Exploratory spatial analysis

**DOI:** 10.1186/1746-6148-6-59

**Published:** 2010-12-30

**Authors:** Zvonimir Poljak, Catherine E Dewey, Thomas Rosendal, Robert M Friendship, Beth Young, Olaf Berke

**Affiliations:** 1 Department of Population Medicine, Ontario Veterinary College, University of Guelph, 50 Stone Road East, Guelph, Ontario, Canada; 2 University of Missouri, 900 East Campus Drive, Columbia, Missouri, 65211, USA

## Abstract

**Background:**

The systemic form of porcine circovirus associated disease (PCVAD), also known as postweaning multisystemic wasting syndrome (PMWS) was initially detected in the early 1990s. Starting in 2004, the Canadian swine industry experienced considerable losses due to PCVAD, concurrent with a shift in genotype of porcine circovirus type 2 (PCV2). Objectives of the current study were to explore spatial characteristics of self-reported PCVAD distribution in Ontario between 2004 and 2008, and to investigate the existence and nature of local spread.

**Results:**

The study included 278 swine herds from a large disease-monitoring project that included porcine reproductive and respiratory syndrome (PRRS) virus-positive herds identified by the diagnostic laboratory, and PRRS virus-negative herds directly from the target population. Herds were included if they had growing pigs present on-site and available geographical coordinates for the sampling site. Furthermore, herds were defined as PCVAD-positive if a producer reported an outbreak of circovirus associated disease, or as PCVAD-negative if no outbreak was noted. Spatial trend was investigated using generalized additive models and time to PCVAD outbreak in a herd using Cox's proportional hazard model; spatial and spatio-temporal clustering was explored using K-functions; and location of most likely spatial and spatio-temporal clusters was investigated using scan statistics. Over the study period, the risk of reporting a PCVAD-positive herd tended to be higher in the eastern part of the province after adjustment for herd PRRS status (*P *= 0.05). This was partly confirmed for spread (Partial *P *< 0.01). Local spread also appeared to exist, as suggested by the tentative (*P *= 0.06) existence of spatio-temporal clustering of PCVAD and detection of a spatio-temporal cluster (*P *= 0.04).

**Conclusions:**

In Ontario, PCVAD has shown a general trend, spreading from east-to-west. We interpret the existence of spatio-temporal clustering as evidence of spatio-temporal aggregation of PCVAD-positive cases above expectations and, together with the existence of spatio-temporal and spatial clusters, as suggestive of apparent local spread of PCVAD. Clustering was detected at small spatial and temporal scales. Other patterns of spread could not be detected; however, survival rates in discrete Ontario zones, as well as a lack of a clear spatial pattern in the most likely spatio-temporal clusters, suggest other between-herd transmission mechanisms.

## Background

Initially reported in early 1990's as post weaning multi-systemic wasting syndrome (PMWS) [1,2], porcine circovirus associated disease (PCVAD) soon became a cause of a major animal-health crisis worldwide. The emergence of PCVAD was remarkable in that it was causally linked with porcine circovirus [3], which, prior to emergence of PCVAD, was believed to be a contaminant of cell cultures [4] with no effect on swine health [5,6]. This virus was subsequently classified as porcine circovirus type 1 [7]. Genetic dissimilarity between the type 1 and the subsequently detected porcine circovirus type 2 (PCV2) is thought to be linked to the difference in virulence between the two types and the consequent emergence of clinical disease. However, detection of PCV2 in historical samples prior to emergence of PCVAD [8,9] is still unexplained. The ubiquitous nature of the virus in swine populations, coupled with common infection (viremia) but variable severity of clinical presentation, as well as the roles of concurrent infectious agents, make the diagnosis of clinical disease in individual animals challenging. The most commonly used set of criteria to define PCVAD is that of Sorden [10], who proposed that all of the following need to be present for confirmation of PCVAD: (i) wasting, weight loss, and respiratory disease, (ii) lymphoid depletion and/or lymphohistiocytic to granulomatous inflammation, typically in lungs or lymphoid tissues, and (iii) PCV2 antigen or nucleic acid associated with microscopic lesions. However, a positive PCVAD diagnosis in individual animals does not necessarily equate to a herd-level problem. It was proposed that a herd was assumed to be facing a substantial PCVAD problem if the level of mortality due to PCVAD is sufficiently higher than historically found in this herd or in area-level averages [3,11].

Starting in 2004, the Canadian swine industry experienced significant losses due to PCVAD. In Ontario, the frequency of reported lesions associated with PCVAD, based on diagnostic submissions to Animal Health Laboratory of the University of Guelph, increased considerably in 2005 and 2006 relative to the period between 2001 and 2004, and a shift in PCV2 genotype was observed [12]. In some herds, mortality in grower-finisher pigs reached as high as 50%. Similar findings were observed in Quebec herds, and ~80% of isolates collected from across Canada during 2005 and 2006 were classified into a previously unreported group, PCV2b [13]. Since then, several vaccine products have been introduced into the market and have generally been effective. Despite research findings concerning the pathogenesis and transmission of PCV2 between animals, data concerning regional spread of PCVAD remain limited. Clustering in space, time, and space and time were identified in Europe, specifically in Denmark [14] and Great Britain [15]. In both studies, the observed pattern of spread between herds was most consistent with introduction of a new infectious agent or a new strain of an agent. Direct transmission through animal movement is believed to be a major contributor [15,16], and previous research has indicated a possible role of seagulls [17] and people [15] as vectors of infection. The pattern of spread consistent with the emergence of a new contagious agent was, however, not always detected. For example, no apparent links between affected herds existed during the early phase of PCVAD (PMWS) emergence in Sweden [18]. Under North American conditions, no results obtained from a large-scale epidemiological study are presently available. The aim of this study was to provide exploratory results for Ontario swine herds, with two specific objectives. **First**, this study aimed to explore the spatial characteristics of self-reported PCVAD distribution and spread in Ontario, and **second**, it aimed to investigate whether a pattern of local spread of PCVAD existed under Ontario conditions, and if it did, to describe its characteristics. For the latter objective, we relied primarily on measures of spatio-temporal clustering (space-time interaction) obtained through space-time K-functions. Briefly, a plot of space-time K-function indicating a relative increase in cases in the vicinity of a case herd would indicate existence of disease spread to neighboring farms and would suggests the characteristics of this spread. Additionally, we investigated measures of purely spatial clustering to investigate aggregation of cases in the proximity of a typical case while ignoring time.

## Results

### Description of the study population

**Figure 1:**
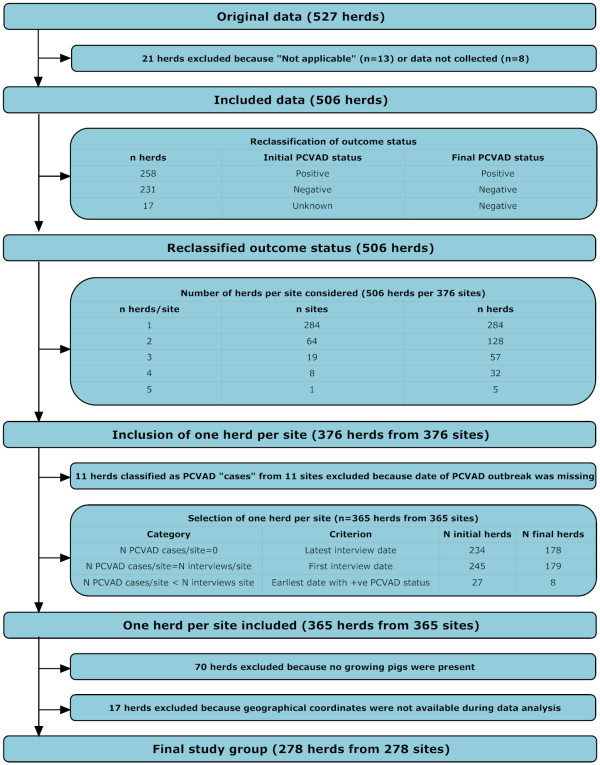
**Selection of swine herds from the original database into a study of the spread of PCVAD in Ontario**.

**Figure 2:**
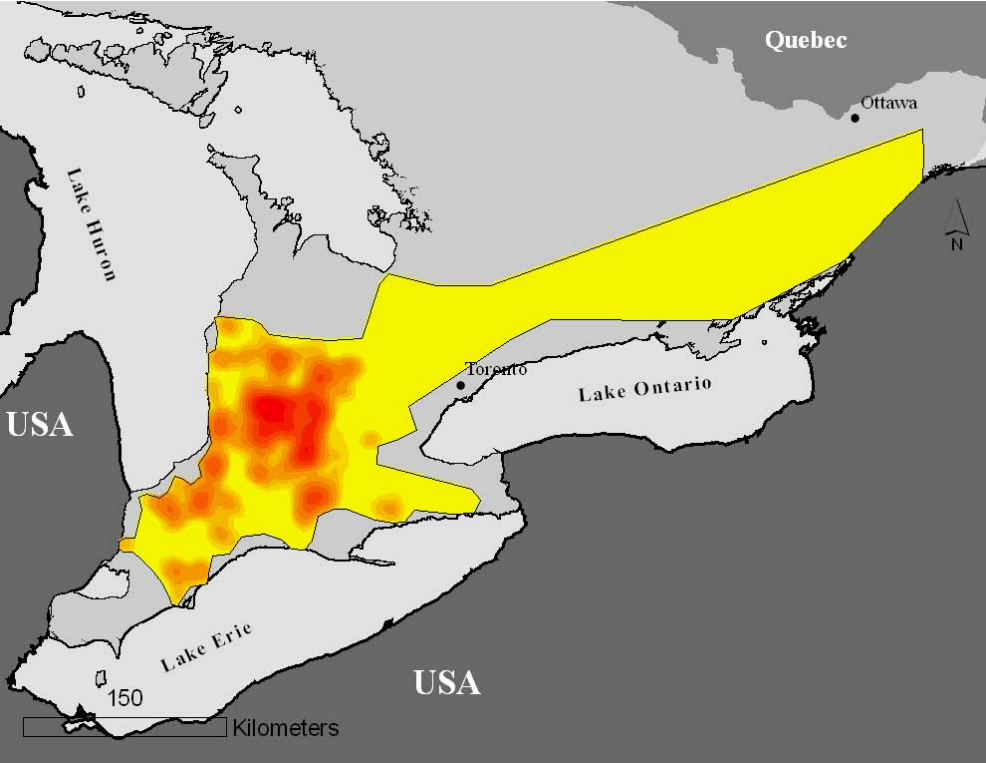
**Location of study area (simplified coloured polygon) within the target area of southern Ontario**. The study area is filled with discrete estimates of herds densities in the study population obtained through kernel smoothing based on Gaussian kernel with fixed bandwidth of 20 km. Estimates are reclassified into 10 categories, and areas with higher intensity of red color represent areas with higher densities of study herds. This density reflects the distribution of swine herds in Ontario. Confidentiality precluded mapping the actual location of herds in the study population.

The study included 278 swine herds, each with a unique premises location, with 170 of these herds declared to be PCVAD-positive. Figure 1 depicts inclusion of herds from the original database into study population. All herds were geographically distributed throughout the study area in the southern part of Ontario (Figure 2). Table 1 contains descriptive statistics of unique Euclidean pairwise distances among all study herds and the Euclidean distance between the nearest neighboring herds. More neighbors were <2 km apart when distance to the nearest neighbor was evaluated than when unique pair-wise distances were evaluated, because repeated values might have been used for distances between the neighboring herds.

**Table 1:** Descriptive statistics of Euclidean distances between all 278 herds and between the nearest neighboring herds in the Ontario herds included in the PCVAD study

	Euclidean distances	
**Statistics**	**All** **herds**	**Nearest** **neighboring** **herds**

Mean (km)	86	5.6
Median (km)	69	3.3
Minimum (km)	0.11	0.11
Maximum (km)	649	97.8
Interquartile range (km)	66	3.9
Number of comparisons <2 km	42	75
Total number of comparisons	38,503	278

Herd demographics are addressed in more detail in an accompanying article [19]. Briefly, 239 herds were positive for porcine reproductive and respiratory (PRRS) virus by polymerase chain reaction (PCR) testing, and 39 were assessed as PRRS-negative by the herd veterinarian. This assessment might have varied between practitioners in terms of diagnostic and sampling approaches, but it implied that the herd was free from PRRS virus shedding and exposure. Descriptive statistics of herds included in analysis are provided in Table 2.

**Table 2:** Descriptive statistics for herds included in the investigation of the PCVAD outbreak in Ontario

	Farm capacity	
	**Nursery** **pigs**^**1**^	**Finisher** **pigs**^**2**^

Mean	1419	1341
Median	990	1200
Interquartile Range	1590	1400
Minimum	50	20
Maximum	9600	6000
n	204	197
		

Farrow-to-finish^1,2 ^(n = 113, 40.6%)		
Farrow-to-grow^1 ^(n = 36, 13.0%)		
Finisher farms^2 ^(n = 71, 25.5%)		
Nursery farms^1 ^(n = 45, 16.2%)		
Wean-to-finish^1,2 ^(n = 13, 4.7%)		

^1,2 ^Calculated only from farms with corresponding superscript

### Spatial and temporal analysis

**Figure 3:**
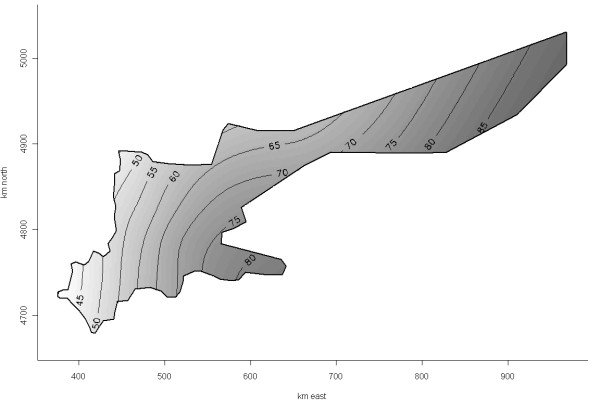
**Expected risk (probability) of PCVAD in the Ontario study population containing 278 herds based on a generalized additive model with a smooth bivariate term for herd locations as the only covariate (*P_s(x, y) _*= 0.16)**. The map shows a relatively simple trend with decreasing risk in the western direction. Note that probabilities depicted on isolines should not be interpreted as prevalence or a risk in the entire population, as it was in part influenced by complex data inclusion mechanism.

**Figure 4:**
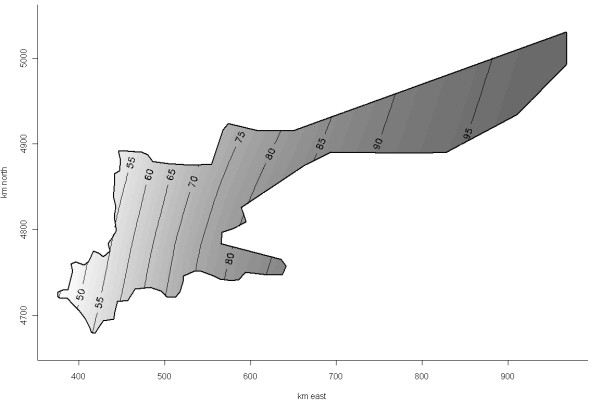
**Expected risk (probability) of PCVAD in the Ontario study population containing 278 herds based on a generalized additive model with a smooth bivariate term for herd locations (*P_s(x, y) _*= 0.05) and porcine reproductive and respiratory syndrome (PRRS) virus herd status (*P *< 0.01)**. The map shows the expected risk in PRRS-positive farms as a simple linear trend with decreasing risk in the western direction.

**Figure 5:**
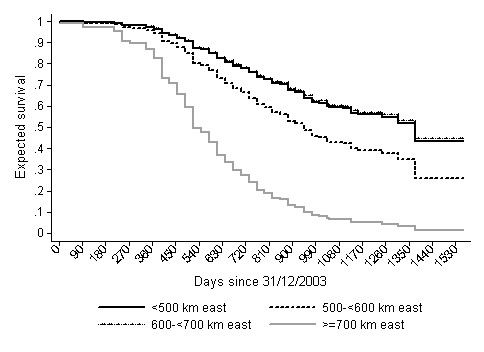
**Expected survival probability for swine herds in four Ontario zones categorized on the basis of easting**. Expectations are for PRRS-positive herds predicted from a Cox's proportional hazard model.

**Figure 6:**
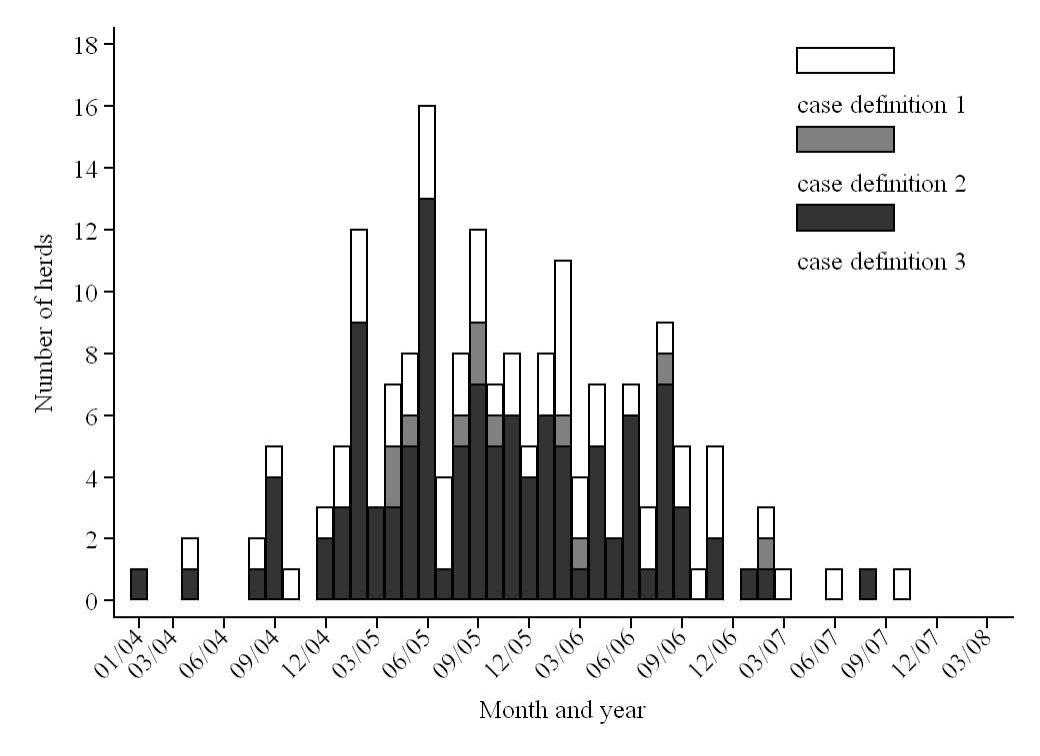
**Number of herds reporting onset of clinical problems due to PCVAD in Ontario between 2004 and 2008 according to three case definitions**. Case definition 1 was based on the producer's recall of a clinical outbreak of PCVAD, case definition 2 was based on the producer's recall of a clinical outbreak of PCVAD and of diagnostic confirmation from the laboratory, and case definition 3 was based on the producer's recall of a clinical outbreak of PCVAD and of diagnostic confirmation from the laboratory and of observation of excessive weight loss in a large number of animals. Note that herds without geographical coordinates were included in this figure, increasing the number of case herds to 179.

The risk of PCVAD positivity appeared to vary over the study area before adjusting for the PRRS virus (PRRSv) status of included farms (generalized additive model [GAM], Figure 3, *P_s(x, y) _*= 0.16), and was significant after this adjustment (GAM, Figure 4, *P _s(x, y) _*= 0.05). The latter was the final GAM model for predicting geographical risk of PCVAD. In April of 2008, when the study ended, the risk of PCVAD - based on this final model - was higher in the eastern areas of Ontario, with a tendency for lower risk in the western parts of the province. The only statistically significant risk factor resulting from univariable analysis was the PRRSv status of the herd. The odds of PCVAD occurring in a herd were 3.4 (95% CI = 1.6, 7.3; *P *< 0.01) times greater in PRRSv-positive farms than in PRRSv-negative herds after adjusting for farm location. Interestingly, the coefficient for PRRSv-positive status increased by 29% on the original (logit) scale after adjusting for trend by including the smooth bivariate function of x (easting) and y (northing) coordinates [s(x, y)]. In addition, herd type was identified as potentially associated with occurrence of PCVAD (*P *= 0.10) during univariable analysis, with nursery operations having lower odds than farrow-to-finish operations (*P *= 0.06). In the final GAM model, no obvious outliers were detected after examination of deviance residuals; however, herds in zone 3 (n = 4) had the highest Cook's D values, i.e., tended to have some disproportionate influence on the model building process. The higher expected risk in the eastern region, based on the final GAM model, was in concordance with the results of the Cox's proportional hazard models. Results of the Cox's model that used four easting zones as categorical covariates suggested that herds in zone 1 of the study area (<500 km east) had a lower hazard than herds in zone 4 (≥700 km east) and zone 2 (500 to <600 km east), but had a hazard equal to that of herds in zone 3 (600 to <700 km east) (Table 3; Figure 5). There was no evidence of non-proportional hazards, as evaluated by scaled Schoenfeld residuals and absence of significance for the time-varying coefficient in the Cox's model. Figure 6 depicts the reported number of PCVAD cases per month according to the three case definitions. The highest number of cases was reported in June of 2005, and 50% of cases were reported by September of 2005.

**Table 3:** Adjusted hazard ratios for herd-level PCVAD status based on classification of easting into four zones§.

Variable	Estimate (HR)	95% CI		*P*
<500 km East (zone 1)	referent	-	-	-
500 to <600 km East (zone 2)	1.58	1.16,	2.15	0.003
600 to <700 km East (zone 3)	0.77	0.19,	3.21	0.724
>700 km East (zone 4)	4.29	1.51,	12.18	0.006
PRRSv status (positive)	1.58	0.92,	2.73	0.097
LR χ^2 ^= 15.68, df = 4, n = 278, *P *= 0.0035, AIC = 1732.9				
No GOF test could be produced for the model with categorical data Global test of proportional hazard assumption (χ^2 ^= 1.69, df = 4, *P *= 0.79)				

^§^The table corresponds to survival curves in Figure 5

**Figure 7:**
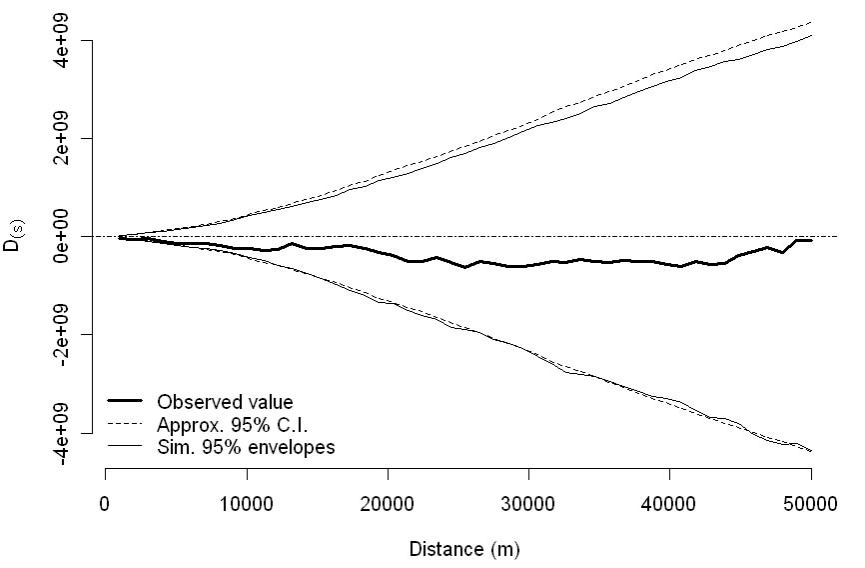
**Observed D function for spatial clustering of PCVAD cases in Ontario, 95% confidence band, and 95% simulation envelope**.

**Figure 8:**
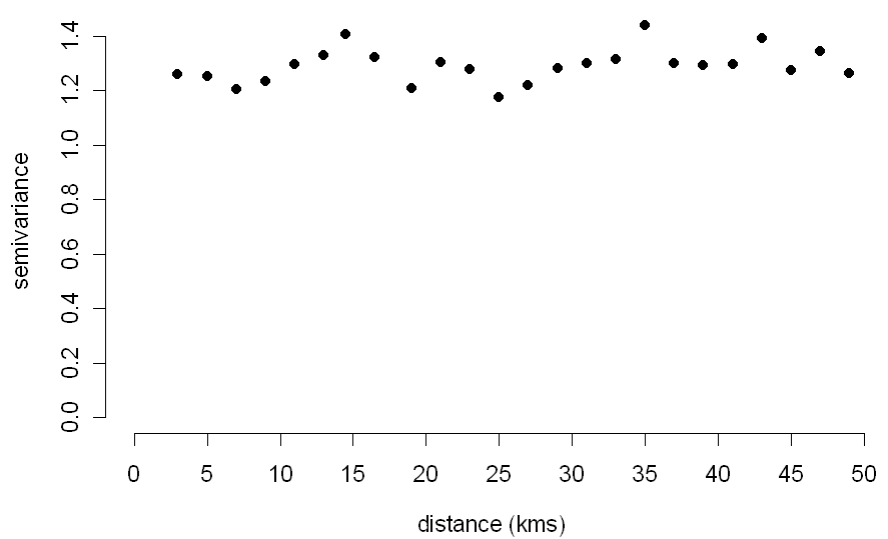
**Empirical semivariogram of deviance residuals obtained from the binomial logistic generalized additive model based on a 2 × 2-km regular grid and geographical coordinates as a smooth bivariate function**.

**Figure 9:**
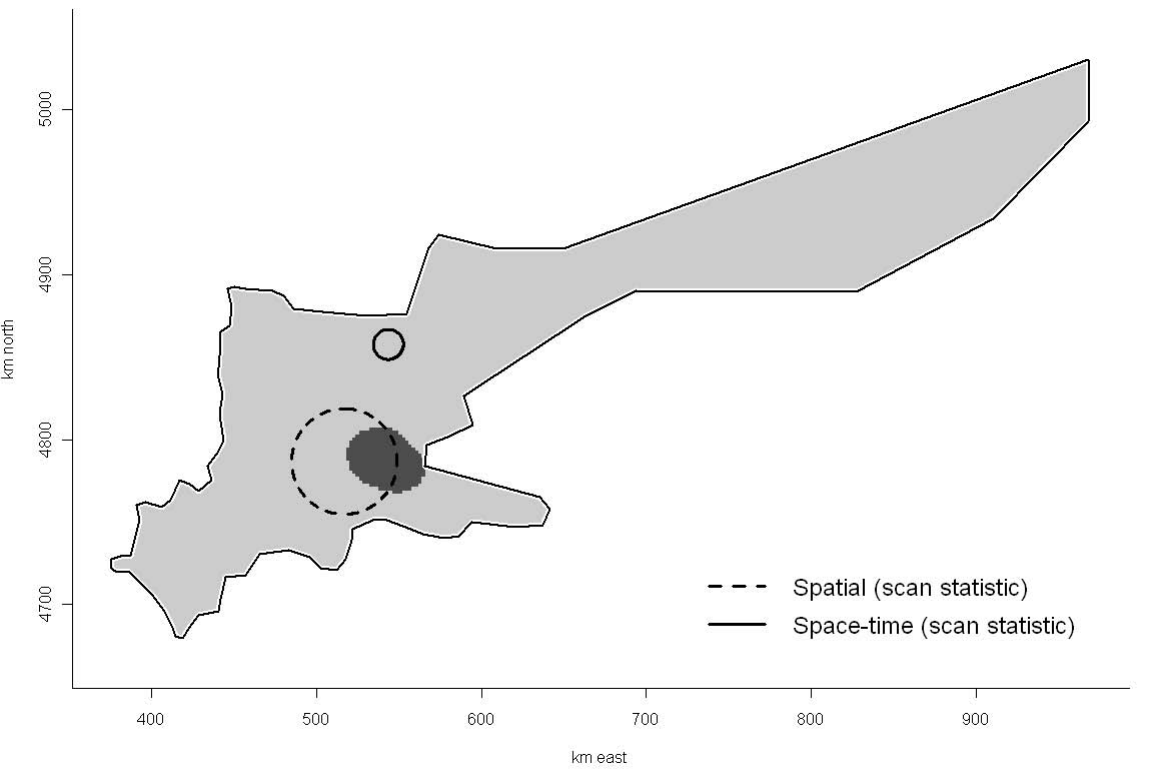
**Location of spatial and spatio-temporal clusters of PCVAD cases detected using three methods**. The dark gray area represents the location of spatial cluster as identified by a generalized additive model based on logistic regression with a smooth bivariate function of x and y coordinates on a 2 × 2-km grid of the southern Ontario study area. The two circles represent the most likely spatial and spatio-temporal cluster detected, using a purely spatial scan statistic and a space-time permutation model, respectively.

Spatial clustering (second-order effect) was not identified in this study when evaluated over the entire study area, as both the D(s) function (simulation *P *= 0.79; Figure 7) and the empirical variogram of deviance residuals obtained from the binomial GAM (Figure 8) failed to suggest any significant spatial clustering. In contrast, the most likely spatial cluster was identified using spatial scan statistics with a centroid in zone 2 (x ~ 517 km; radius = 32 km, RR = 1.5, *P *= 0.04; Figure 9 ["Spatial"]). The spatial cluster identified by GAM partly overlapped the spatial cluster detected by the spatial scan statistic, suggesting involvement of herds in the same general area in this cluster. We did not further investigate characteristics of herds included in this high-risk area.

### Spatio-temporal analysis

**Figure 10:**
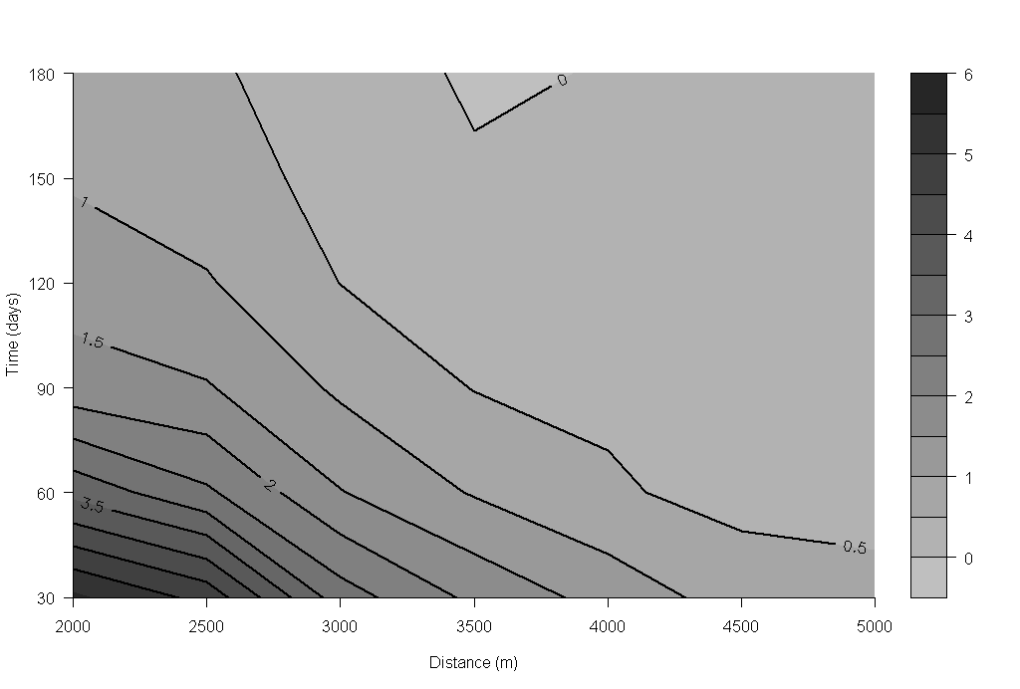
**Spatio-temporal K-function of PCVAD spread under Ontario conditions**. Areas in the lower left corner, representing neighborhoods close in space and time to an outbreak farm, pose higher risk for farms in that area. This is indicated by darker shades of gray and lines above the proportional increase in risk of 1.

**Figure 11:**
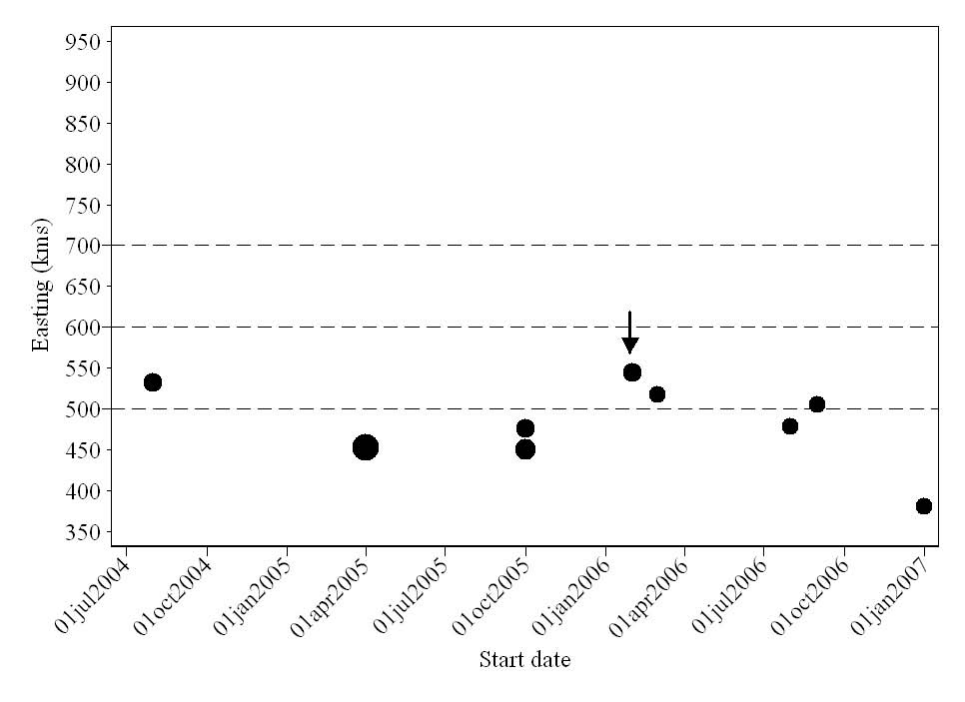
**Scatterplot of all reported spatio-temporal clusters of reported PCVAD cases identified by a space-time permutation model in the study area between January 2004 and April 2007**. The y axis represents easting of the cluster centroid, and the x axis represents the start date. Size of dots is proportional to cluster size. Gridlines represent categorization of the study area in four different zones with respect to easting, in agreement with classification of zones in Figure 5. The most likely cluster is identified by arrow.

Examination of the spatio-temporal K-function revealed a space-time interaction in the data, originating primarily from small-scale clustering in space and/or time (simulation *P *= 0.06). The contour plot of the D_0_(s, t) suggested a proportional increase in risk attributable to space-time interaction; herds that were closer than ~ 4.5 km to an incident PCVAD-positive herd seemed to be under increased risk (relative increase >1) of a PCVAD-outbreak over the next 5 mo (Figure 10). As the distance (in time and space) to the incident herd decreased, the excess risk of developing PCVAD increased to reach a maximum at approximately 2 km of spatial and 1 mo of temporal distance. Interestingly, this risk seemed to be lower for herds located <2 km apart (data not shown). Since only a few distances between cases were <2 km, we decided to evaluate our spatio-temporal K-function starting at 2 km. Investigations at larger spatial and temporal scales yielded similar conclusions (data not shown). In addition, one significant space-time cluster was detected, with a radius of 9.1 km and a duration of 1 mo starting in February 2006 and involving four herds (*P *= 0.04; Figure 9 ["Space-time"]). Although no secondary spatio-temporal clusters (*P *< 0.3, n = 8) reached statistical significance, their exploration yielded some insight into the development of the epidemic. In all but one case, the duration of likely space-time clusters was ≤ 3 mo, the radius was <30 km, and the number of farms included was ≤ 5. This descriptive information further supported the clustering of incident PCVAD cases on a small spatial and temporal scale depicted by D_0_(s, t). Furthermore, Figure 11 contains a scatterplot of all likely spatio-temporal clusters with kilometers of easting on the y axis, start date on the x axis, and dot size proportional to the number of herds in the cluster. The scatterplot is further divided into four zones according to categories of easting (gridlines of the y axis) previously used as discrete levels in survival analysis. It is evident that all likely clusters were detected in zones <600 km east, with the highest density of the swine population in Ontario. Moreover, there was a lack of clear correlation between easting of the most likely outbreaks and their starting dates, in contrast to the correlation between these parameters at the herd level. Thus, it seems that these apparent spatio-temporal clusters were generated by a process with no clear linear evolution over a geographical area in the x direction.

## Discussion

Collectively, the results of this analysis suggest three major findings: (i) existence of spatial variation in risk, (ii) existence of apparent local spread, and (iii) possible existence of other mechanisms of spread.

Spatial variation in PCVAD risk between the eastern and western part of the study area existed (as suggested by the GAM) and there was a general east-to-west trend in PCVAD spread (as suggested by Cox's proportional hazard model). Similar findings of directionality were observed in other studies that investigated outbreaks at the national or regional level. Woodbine et al [15], investigating outbreaks in Great Britain, reported that PCVAD outbreaks were first observed in the south of England, progressing toward the west and north. In Denmark, Vigre et al [14] reported the existence of two large spatial clusters of positive herds during an initial 2-y period of disease occurrence, and although geographical variability in the occurrence of disease existed, directionality was not apparent from the results, nor was it discussed. In the early phases of the outbreak, geographical variation was, as expected, found in other study regions [17,18]. The observed directionality was contrary to the dominant winds in Ontario, but it was in close concordance with one of the "principal routes" of the Atlantic bird flyway http://www.birdnature.com/upperatlantic.html through the study area. In previous studies of PCVAD emergence, it was suggested that southern black-backed seagulls were possible mechanical vectors of indirect spread of a causative agent [17]. Data collected in this study did not allow investigation of birds as mechanical or biological vectors. Moreover, the directional spread pattern might also be linked with other direct or indirect mechanisms. Finally, it cannot be completely excluded that farmers in eastern Ontario have different social contacts or sources of information than producers in the western part of the province, and would consequently have a different sensitivity or threshold to declare herds PCVAD-positive. Although only a few herds were sampled from this region, these herds did not appear to be outliers in either regression model. In addition, existence of differences in terms of social contacts cannot explain the apparent difference in spatial risk between herds located in zone 2 and zone 1, where the identical spatial trend was observed. Although the PCVAD risk surface, generated by GAM, suggested the existence of this directionality, we caution the reader that the actual numerical risk estimates are not directly interpretable as estimates of prevalence or of incidence risk in an area, particularly in the unadjusted GAM, because of the initial sampling and subsequent inclusion criteria (which might influence the estimate of the intercept and therefore the entire risk surface).

The lack of evidence of purely spatial clustering (ie, tendency of disease-positive farms to occur near other disease-positive farms - spatial autocorrelation), was not in agreement with detection of a significant purely spatial cluster, for two possible reasons. First, in theory, clusters of disease-positive cases could occur even in the absence of interaction (spread) between farms. A typical example would be environmental factors impacting herds independently. Thus, clusters could be detected even if spatial autocorrelation does not exist. Second, the methods used to identify the existence of spatial clusters might have higher sensitivity than those used to identify spatial clustering, at least as we used these methods in the study. We used the latter set of methods over the entire study area, in contrast to the use of similar methods (difference in K-functions) in specific regions to investigate other swine diseases [20]. The rationale for our approach was that we had no predefined local area of interest prior to analysis, and we used methods intended to accommodate the heterogeneous distribution of the study population.

Apparent local spread (neighborhood spread) existed, as suggested by the existence of clustering and clusters on a small spatial and temporal scale. During the 2001 foot-and-mouth (FMD) epidemic in the UK the term "local spread" was used in a field when a new FMD case was identified within a 3 km of a previous FMD case, and contact with a more distant source of FMD could not be determined [21]. This description of local spread was more in line with the field investigation of outbreaks on a case-by-case basis, whereas our approach to investigating local spread was comparable to the analytical approach taken by Picado et al [22]. Similar findings for PCVAD clustering were reported in Denmark [14] and subsequently in Great Britain [15], although the scale of spatio-temporal clustering in the latter study seemed to be larger than in this study. High pig-farm density in the neighborhood was identified as a risk factor in other studies as well [23]. A pattern of local spread could be the consequence of a combination of different mechanisms, including direct, indirect, and airborne transmission of an agent identified as a necessary cause (PCV2) or a component cause (eg. PRRSv). These mechanisms could not be easily discriminated using the data at hand. While the possibilities of direct and indirect transmission are discussed in previous reports, data concerning airborne spread are scant. Recently, Verreault et al [24] reported detection of PCV2 genome copies in the air of confined finisher barns in concentrations of up to 10^7 ^copies/m^3^. The significance of this finding is yet to be elucidated. Although none of the secondary likely spatio-temporal clusters were statistically significant, they exhibited some common characteristics. They were observed in zones 1 and 2, where the majority of pig herds in the study population were located, involved a small number of herds, and were short in duration and limited in geographical extent. The latter two observations were in agreement with the results of the spatio-temporal K-function. However, given the number of herds in this study, statistical evidence to support existence of apparent local spread was relatively weak. This could be a consequence of non-differential misclassification due to the non-specific case definition; alternatively, local spread may have been only one component of disease transmission, or perhaps that apparent local spread was confounded by clustering of farms under the same ownerships in the same geographical area. Thus, the results of this study suggest that farms in the vicinity of those experiencing PCVAD outbreaks were at increased risk of experiencing outbreaks for months. Recommendations based on these findings were modified once other factors were taken into account. The results and methodological approaches taken are reported in an accompanying article [19].

At least two inconsistencies in the results suggest the existence of patterns of spread other than apparent local spread. Firstly, while the existence of a spatial trend in the study region was confirmed in various analyses, the most likely spatio-temporal clusters occurred almost haphazardly in x direction and time (Figure 11). This suggests that disease did not spread as a sequence of geographically connected outbreaks in the east-west direction. Secondly, the expected time to outbreak in herds located in zone 3 was equivalent to that in zone 1, while herds located in zone 3 had the largest residuals and were influential points in the GAMs. It is possible that herds in this area were more isolated from the major centers of swine production than herds in the other three areas. This "isolation" could be with respect to direct or indirect potential sources of infection. The infectious agent could have also spread via other transmission pathways, primarily through movements within swine production companies and their suppliers. Bigras-Poulin et al [25] showed that patterns of swine movement in Denmark had the topology of a scale-free network. Theoretically, much lower transmission probabilities on scale-free networks are sufficient to spread or maintain "infection" on these networks than under the assumption of a homogeneous mixing [26]. Recently, Firth et al [27], using PCV2 sequence data, reported several significant diffusion pathways for this agent between different continents and between different countries. The pathways identified were similar to the trade patterns of swine. Despite the limitations inherent to such data (ie, reporting time of sequence data) and study design (ecological study), the conclusions suggest that live-animal movements contributed to disease spread. Woodbine et al [15] identified purchase of breeding stock as a risk factor for herd outbreaks in the early phase of the PCVAD epidemics in Great Britain. In this respect, it is possible that Ontario herds in zone 4 (>700 km east) interacted more with herds in the neighboring province (Quebec) than with other Ontario herds. In contrast, herds in zone 3 (600 to <700 km east) were likely sufficiently distant in both geographical and road distances from centers of intensive production in the highly swine-dense areas of Ontario and Quebec to decrease the frequency of direct contacts (through animals) and indirect contacts (through fomites) with herds from these centers. This is consistent with the findings of Madec et al [16], who argued that contact between pigs is the main route of transmission for PCVAD. In agreement with direct contact as a major contributor to PCVAD spread, ownerships as proxy measurements for network memberships were investigated in the accompanying article [19]. However, regardless of whether spread through networks occurred or not, it should be stressed that detailed investigation of production-disease epidemics in intensive production systems that prevail today in industrialized countries is challenging if appropriate movement and contact information is not readily available. Despite these limitations, results gained through this exploratory analysis support the spread of the infectious agent in accordance with other studies [14,15].

Generalized additive models were useful with respect to investigating different aspects of PCVAD. Some advantages of the trend surface produced by the GAM were that it could be easily adjusted for risk factors, and residuals and influential values could be examined. In addition, deviance residuals could be produced from the model using binomial (aggregated) data to construct an empirical variogram. This approach has the advantage of evaluating second-order effects (clustering) after accounting for the first-order effects (geographical trends). However, this aggregation of herd-level (Bernoulli) data to areal (binomial) data (in our case using a 2 × 2-km grid) introduced two limitations. First, herd-level covariates could not be used in a model unless they were aggregated to the same level as the outcome. Second, as in any logistic regression model, a reasonable number of observations in each covariate pattern is needed to achieve desirable properties of residuals [28]. For the aggregation used in this study, this could be attained only if the density of sampled points in a grid was high or the area of the grid used to collapse the data was large. Finally, the GAM further offered the opportunity to investigate the location and significance of local clusters. This approach was based on previously published methodology [29]. In this study, both approaches identified spatial clusters in the same region, although the location and the extent of the two high risk areas only partially agreed. We opted to use the GAM approach to identify spatial trend and risk factors in a purely spatial model, as opposed to the logistic regression based on fully Bayesian linear mixed model with spatially structured residuals (for example, see Peng et al [30]). Although performance of the two approaches was not compared, one of the advantages of the GAM approach over a generalized linear model (Bayesian or otherwise) is that the GAM allows for fitting a very flexible trend surface for spatial point data.

This study has several important limitations. First, the study population represented a biased sample of the Ontario swine population at that time. One explicit inclusion criterion was that the referent veterinarian was to be a member of the Ontario Association of Swine Veterinarians (OASV), and information was shared through OASV's e-mail list. This group of veterinarians consults for most commercial swine herds in Ontario. Premises holding small numbers of pigs may be under-represented in the study population because these farmers may be less likely to ask for the services of a veterinarian. We believe that this increased the accuracy of diagnosis, because clinical signs suggestive of systemic PCVAD are easier to observe in large populations. Moreover, we believe that this study was not biased with respect to geographic location, because two diagnostic laboratories that perform PRRSv testing are located within a 30-km Euclidean distance, and some samples from the alternate diagnostic laboratory were eventually included in the study when PRRSv sequencing at no cost was offered as an incentive to participate in the study. Therefore, we believe most eligible herds were included in the study. We cannot provide complete statistics on this, since inclusion of a herd was the responsibility of not only the research team (e.g., whether an interview could be arranged), but also of the veterinarian (e.g., whether samples should be submitted, whether the premise and the case submission should be included in the study). It was anticipated that a comparatively high percentage of PRRSv-negative herds would be identified in eastern Ontario, as the density of swine herds in this region is much lower than in western or southern Ontario. We included the PRRSv status of a herd at the time of sampling in an attempt to adjust analytically for such stratification, when the risk surface was produced. Results of adjusted analysis suggested that the spatial trend was simpler and more suggestive of directionality than the expectations from the unadjusted analysis. A mapping approach using GAM was very useful in this respect.

Bias might also influence detection of clustering and clusters. We believe that the impact of this bias is small for the following reasons: (i) PCVAD-positive and negative herds were selected from the same target population using the same selection mechanism, and (ii) premises with multiple herds included over time were aggregated to only one, the most relevant herd. Since PRRSv-positive status is associated with higher odds of being a PCVAD-positive herd, potential spread of PRRSv or a specific PRRSv genotype might have positively contributed to measures of clustering for PCVAD.

The second important limitation of the study is a non-specific case definition that depended on the memories of the producers. The extent and influence of potential misclassification bias is difficult to ascertain. However, results of the several initial analyses that were based on more stringent criteria to define a case (e.g., was PCVAD verified by laboratory testing) confirmed the same conclusions with respect to temporal (Figure 6) and spatial trends (data not shown). Consequently, we decided to use the maximum amount of data available. In addition, herd-level diagnosis of PCVAD is difficult, as even identifying PCVAD in individual pigs does not necessarily equate to a clinical problem in the herd. Herd-level diagnosis of PCVAD is ultimately based on mortality rate in the growing pig, which, in the absence of regular electronic monitoring, has to rely on the operator's assessment. The reader should also be aware that under the "Circovirus Inoculation Program" [31], swine producers could qualify for compensation for diagnostic testing and vaccination for PCV2 performed between March 1, 2006, and December 31, 2008. This likely increased diagnostic testing and confidence in declaring PCVAD status of a herd. Finally, within a region, spatially referenced data about the spread of a disease such as PCVAD is difficult to obtain under current conditions.

Third, one could also argue that a large number of herds were excluded from our analysis. However, the purpose of this step was to eventually have only one, the most relevant, data point per premises - representing the first occurrence of a disease at that site.

Fourth, introduction and uptake of effective vaccines might have had an undue influence on our results. At least one commercial vaccine was introduced in Canada and the USA starting in late March and April of 2006 [32,33] under conditional license. Due to limited supply, it was initially distributed to herds with documented outbreaks. At the time of vaccine introduction, the peak of the outbreak in this study population had already occurred in June of 2005 (Figure 6). Thus, the most likely impact of this intervention on the results of the study was to diminish any effects identified. This also illustrates a need to evaluate possible time-varying patterns of disease spread. Despite its limitations, we believe our investigation will provide additional information about this important disease, particularly under North American farming conditions.

## Conclusions

In conclusion, a pattern of spread consistent with the emergence of an infectious agent was supported by a spatial trend (pattern). Over the study period, the risk of reporting a PCVAD-positive herd seemed to be higher in the eastern part of the province. This directionality was partly confirmed for spread using survival analysis. A pattern of local spread also appeared to exist, as evidenced by spatio-temporal clustering of PCVAD and existence of spatio-temporal clusters. This second-order effect was based primarily on clustering on the small spatial and temporal scale. The spatial trend and clustering could be due to direct or indirect mechanisms of spread and were not explored further in this analysis. Other patterns of spread were suggested by the PCVAD failure risk in zone 3, that was indistinguishable from the PCVAD failure risk in zone 1 (far west part of southern Ontario), and a lack of clear geographical directionality of the most likely apparent spatio-temporal clusters. Generalized additive models as used in this study can be a useful complement to exploratory spatial analysis.

## Methods

Analyses were conducted in three stages to document the spatial, temporal, and spatio-temporal characteristics of the data. In the first stage, we investigated spatial trend, disease clustering, and clusters using a combination of generalized additive models (GAM), spatial K-functions, and scan statistics. In the second stage, the epidemic curve was inspected visually and factors associated with herd breakdown were evaluated using Cox's proportional hazard model. In the third stage, space-time clustering was evaluated using a combination of space-time K-functions and scan statistics based on a space-time permutation model.

### Data sources

The hierarchy of populations in this study could be briefly explained in the following way. The target population for this study was the population of swine herds in Ontario. The source population was herds that had members of the Ontario Association of Swine Veterinarians (OASV) as herd veterinarians and had at least one submission to a diagnostic laboratory between 2004 and 2007 if they were designated as PRRSv-positive. Herds were eligible for inclusion if growing pigs between weaning and market weight were present on the premises and if they had full information required to define PCVAD status, location, and time of outbreak if classified as PCVAD-positive. In addition, only one herd per premises was eligible for inclusion according to specific criteria.

Specifically, the 278 herds included in this study were a subset of herds included in the Ontario PRRSv monitoring project. The objective of this larger project was to map occurrence of different PRRSv genotypes in Ontario in order to provide monitoring and disease-control information to the Ontario swine industry. Herds were included in this larger project using two methods. First, PRRSv-positive herds across Ontario were included after identifying owners of farms with PRRSv PCR-positive submissions to a diagnostic laboratory (Animal Health Laboratory [AHL]; University of Guelph, Ontario, Canada) between 2004 and 2007 who agreed to participate in the study (n = 466). Second, cooperating members of the OASV identified herds that were PRRSv-negative during the study period, using their own historical diagnostic data (n = 61). Producers having herds from either PRRSv-infection stratum were interviewed by phone by one of the three interviewers and asked questions about occurrence of a PCVAD outbreak in their herd (referred to as "circovirus problems" and additionally prompted as "PMWS" during the interview). The telephone interviewee was the person most familiar with animal handling within a specific herd at the time of the interview. In some cases, this required multiple phone calls.

The inclusion criteria for this study were (i) having nursery or finisher pigs on the premises, (ii) only one herd per premise wherever multiple herds (case submissions) were available per premise, and (iii) available geographical coordinates. The inclusion process is presented in Figure 1. Of the 527 herds available, 21 herds that had a missing answer on PCVAD status were excluded from subsequent analysis; in eight herds, data on PCVAD were not collected, as a subsequent herd visit with more thorough diagnostic follow-up was arranged during the interview; and in 13 herds, the producer responded that the question was not applicable. In yet another 17 herds, the producer did not know whether PCVAD had occurred in the herd, and this was coded as a negative response with the rationale that a producer would recall a devastating outbreak of a new disease in her/his herd. In 231 herds, the answer to the question on occurrence of problems related to circovirus was "no." In 258 herds, the answer was "yes," and these herds were considered PCVAD cases during this data-processing step. Overall, this yielded 506 herds sampled from 376 sites, with 284 unique herds within sites, 64 sites interviewed twice, 19 sites interviewed three times, eight sites assessed four times, and one site assessed five times. Only one herd per site was included, using three criteria. First, if the number of PCVAD cases (herds) per site was 0, the herd with the latest interview date was selected in an attempt to maximize follow-up time per site (n = 234 herds; n = 178 sites [161 single sites, 16 selected from repeated sites, and one site with three available records]). Second, if the number of PCVAD cases per site was equal to the number of interviews, the observation with the earliest interview date was selected in an attempt to reduce recall bias for positive herds (n = 245 herds; n = 233 herds with non-missing dates, n = 179 sites, [165 single sites, 13 selected from repeated sites, and one site with three available records]). During this process, 14 herds from 11 sites were excluded because they had had a PCVAD outbreak, but the producer could not recall the date of its onset. Third, if the number of PCVAD-positive herds per site was at least one, but lower than the number of herds included per site, then only herds with a reported outbreak were selected for further processing and the one with the earliest date was included (n = 27 herds, n = 13 positive herds, n = 8 sites). Overall, 365 herds on unique sites were available, but only 295 were included because they had either a nursery production phase, or a finisher production phase, or both. This dataset was further reduced to 278 sites, eliminating 17 because geographical coordinates were not available for these sites when the data were analyzed.

Producers were asked several questions related to PCVAD, including whether the premises had ever experienced an outbreak of PCVAD (case definition 1), whether additionally this outbreak was confirmed by laboratory testing (case definition 2), whether additionally observation of excessive weight loss in large number of animals was noted (case definition 3), and date of onset, typically recorded within a month accuracy. The interview period was January 2006 to April 2008. In addition to epidemiological information, the spatial location of each premises was obtained from the Ontario Pork Marketing Board or by georeferencing the barn address. Ontario Pork maintains a database of swine premises in Ontario, and we received a set of coordinates (as longitude and latitude) for each premises that was identified as a site of interest (sampling site and site for which epidemiological information was collected). If this information was not available, researchers manually searched aerial photographs of Ontario identifying the most likely premises using the site address and taking point coordinates (longitude and latitude) at the location that appeared to be the entrance door. In addition, farm existence and farm type on the sampling site were visually confirmed regardless of the source of information for coordinates. An included herd was defined as PCVAD-positive if the producer reported an outbreak of PCVAD on the premises; otherwise, the herd was considered PCVAD-negative. Categories were assigned after three different case definitions were evaluated and results of simple descriptive and inferential analyses, as well as initial geographical analysis, yielded qualitatively identical conclusions. For example, Figure 6 depicts an epidemic curve based on three different case definitions. Thus, our decision was to select the case definition that would include all available herds (case definition 1), thereby maximizing the information available in the dataset, in line with the exploratory nature of this analysis.

### Data management

Data obtained from the telephone interviews were entered into a hierarchical database (Microsoft Access 2003) with logical checks to ensure accuracy. Data were entered by three researchers who participated in the interviews. Data processing was performed using commercial software (Stata 10 SE). Geographical coordinates were projected into a projected coordinates system (UTM NAD Zone 17 N; ArcInfo 9.1) with meters of easting (x) and northing (y) as the units.

### Spatial analysis

The study area was defined as an irregular polygon obtained manually, so that study sites were completely included within the study area (splancs, R 2.8). Actual boundaries of the polygon were based on subjective visual assessment. After mapping marked point data, a large-scale analysis of spatial trend (first-order effect) was performed using generalized additive models (GAMs; (mgcv, R 2.8; [34])), with resulting risk surfaces. Risk surface was obtained using the following steps.

First, the basic Bernoulli regression model was fitted to the observed data: logit(PCVAD = 1) = *β*_0 _+ *s*(*x*, *y*), where *s *refers to the smooth bivariate function of x and y coordinates. The smooth function was based on thin-plate regression splines (TPRS) [34], and parameters that were used during modeling allowed for potentially high complexity of the surface (maximum *k *= 100). Parameter *k *is the maximum allowed choice for the basis dimensions (TPRS in our case) and is equivalent to setting the maximum possible degrees of freedom for each term in a model. The actual effective degrees of freedom (edf) for the term are estimated from the data using smoothing parameter selection criteria, with *k*-1 representing the upper limit of the estimate [34].

Second, this basic model was expanded by inclusion of herd-level covariates identified as significant at *P *< 0.10 during the univariable logistic regression modeling. Factors that were evaluated included (i) herd type, (ii) total number of pigs on site, and (iii) PRRSv status.

Third, after fitting each model, the expected values on a probability scale were obtained on a regular 2 × 2-km grid of the study area. A similar approach was reported elsewhere to explore the spatial distribution of dengue fever in Brazil [35].

Existence of spatial autocorrelation (disease clustering or second-order effect) was evaluated using a difference between K-functions [36] for PCVAD-positive and PCVAD-negative herds [D(s)] [37]. The D(s) was evaluated over a 50-km distance in sequential steps of 1 km. The simulation envelope for D(s) was constructed using 999 random permutations of case labels to all locations with a resulting Monte-Carlo based *P*-value and 95% simulation envelope constructed from the 2.5^th ^and 97.5^th ^percentiles of simulated values for D(s) (splancs, R 2.8, [38]). The approximate 95% confidence intervals on D(s) were also constructed (splancs, R 2.8). In addition, binomial logistic regression using the GAM approach with s(x, y) based on centroids of a 2 × 2-km regular grid of the study area (instead of actual farm locations) was additionally fitted, and the empirical semivariogram of deviance residuals was examined for evidence of the second-order effect as described elsewhere (mgcv, R 2.8, [34]).

The existence and location of the most likely spatial cluster of PCVAD-positive herds were determined using two different methods. First, the scan statistic, based on a purely spatial Bernoulli model [39] using default parameters, was used in available freeware (SaTScan [40]). Second, the unadjusted GAM, based on the actual farm locations (not grid centroids), was used to generate log odds ratio for each cell of the 2 × 2-km grid of the study area (cell). Standard error of the estimate in each cell was used to calculate the approximate 95% confidence interval and the location of a cell where the lower limit of the 95% confidence interval for odds ratio was >1 was considered to be a part of the spatial cluster. The use of GAM for detection of spatial clusters is reported elsewhere [29].

### Temporal analysis

Since producers were interviewed over the extended period of time during which the outbreak of PCVAD was progressing, there was a concern that on some premises, outbreaks might have occurred after the interview date. Thus, the importance of the large-scale effect was examined using Cox's regression model where demographic factors identical to the ones used in logistic regression (GAM) were examined. Time-to-event was determined as the number of days between December 31, 2003, and the date of a reported PCVAD outbreak for positive herds, or the number of days until the interview date for PCVAD-negative herds, when negative herds were considered censored. For the survival analysis, easting was used as a categorical variable after it was categorized into four zones (<500 km east [zone 1], 500 to <600 km east [zone 2], 600 to <700 km east [zone 3], and ï‚³ 700 km east [zone 4]). This decision was based on the ease of interpretation and used steps of 100 km in the most pig-dense area. Overall fit and survival model assumptions were evaluated as reported elsewhere [41].

### Spatio-temporal analysis

The spatio-temporal K-function [42] and its relative contour plot [D_0_(s, t)] were used to investigate the existence and significance of space-time interaction over 5 km of spatial distance and 6 months of time. A value of D_0_(s, t) could be interpreted as a proportional increase in the cumulative number of cases within a distance s and time t of a randomly selected case that is attributable to space-time interaction. A value of D_0_(s, t) > 0 suggests space-time interaction, with a value of D_0_(s, t) > 1 indicating a relative increase of 2 times the expected number of cases; the latter cutpoint has been used as an important decision point in previous studies [22,43]. In addition, a scan statistic, based on the space-time permutation scan statistic [44], was used to detect the location and occurrence of the most likely spatio-temporal clusters. The latter analysis was based on default parameters and 999 permutations using month as the temporal unit of interest. Primary and secondary spatio-temporal clusters have been investigated for their characteristics.

## Authors' contributions

ZP participated in study design, partly processed the data, statistically analyzed the data and interpreted results, and wrote the initial draft of the manuscript. CED proposed and acquired funding for the large monitoring program, participated in study design, developed the questionnaire, and commented on the initial draft. TR participated in study design, questionnaire development, and data processing and commented on the initial draft. RMF participated in questionnaire development and commented on the initial draft. BY collected data and provided comments on the initial draft. OB commented on the spatial analyses and on the initial draft. All authors read and approved the final version.
